# Two-year radiologic assessment of the Pinnacle cup—a migration analysis with EBRA

**DOI:** 10.1007/s00402-020-03648-4

**Published:** 2020-10-30

**Authors:** Dietmar Dammerer, Alexander Ruzicka, Philipp Blum, David Putzer, Maximilian Liebsch, Julian Lair, Martin Thaler

**Affiliations:** 1 Department of Orthopaedics and Traumatology, Medical University of Innsbruck, Anichstrasse 35, 6020 Innsbruck, Austria; 2 Department of Experimental Orthopedics, Medical University of Innsbruck, Sonnenburgstr. 16, 6020 Innsbruck, Austria

**Keywords:** Cup migration, Total hip arthroplasty, Cementless, Einzel-bild-röntgen-analyse (EBRA)

## Abstract

**Introduction:**

The most common cause of failure in total hip arthroplasty (THA) is aseptic loosening. Uncemented cup migration analysis by EBRA (Einzel-Bild-Roentgen-Analyse) has shown to be a good predictive indicator for early implant failure if the cup migrates more than 1 milimeter (mm) within the first 2 years after surgery. In this study, we investigated the migration behaviour of an uncemented press-fit cup after 2 years follow-up.

**Materials and methods:**

Applying a retrospective study design, we reviewed all consecutive patients who received an uncemented press-fit cup at our Department between 2013 and 2018. A total of 484 patients were identified. We reviewed medical histories and performed radiological measurements using EBRA-Cup software. EBRA measurements and statistical investigations were performed by two independent investigators.

**Results:**

A total of 165 cups in 159 patients (female: 90; male: 69) met our inclusion criteria. Mean age at surgery was 66.7 (range 18.4–90.5) years. EBRA migration analysis showed a mean total migration of 0.7 mm (range 0.0–6.3) over our follow-up period of 2 years. Of the investigated cups, 53.2% showed less than 1 mm migration in the investigated follow-up period.

**Conclusion:**

In conclusion, the Pinnacle cup used in our study provides low mean migration at final follow-up. Based on the assumption of secondary stabilization, good long-term outcome of the Pinnacle cup can be expected.

**Trial registration number and date of registration:**

Number: 20181024-1875; Date: 2018-09-20

## Introduction

The most common cause of failure in total hip arthroplasty (THA) is aseptic loosening [1]. According to the literature, cup migration of more than 1 milimeter (mm) within the first 2 years after surgery is a well-established risk factor for early implant failure [2–4]. Previously published studies of uncemented cup migration investigated by means of EBRA (Einzel-Bild-Roentgen-Analyse) have shown this migration to be a good predictive indicator and threshold for later aseptic loosening [5–8].

EBRA is a computer-assisted method for measuring the migration of acetabular cups using standard anterior–posterior (ap) pelvic radiographs without requiring additional means at exposure (e.g. ball markers) [9]. It has proven accuracy and sensitivity in detecting migration of more than 1 mm as compared to RSA (Roentgen stereophotogrammetric analysis) [10–12].

According to the Australian Orthopaedic Association National Joint Replacement Registry, 6333 Pinnacle acetabular cups were implanted in 2018, making it the second most used cup in primary THA [13]. The Pinnacle acetabular cup system is a cementless cup prosthesis that can combine a polyethylene or ceramic inlay with a metal or ceramic head. Being a cementless system, cup fixation is achieved with the press-fit method [14]. Reaming the acetabulum can be done by under-reaming, over-reaming or line-to-line reaming. The method used is chosen by the surgeon and subject to patient age and bone quality.

In the present study, we used EBRA to investigate the clinical results and the migration behavior of the uncemented Pinnacle cup after a mean follow-up of 2 years.

## Materials and methods

The study was approved by the local ethics committee (Medical University of Innsbruck, Austria, Europe). We applied a retrospective study design and reviewed all consecutive patients who received a Pinnacle cup at our Department between 2013 and 2018. During this time, a total of 484 Pinnacle cups were implanted as part of a primary THA.

We examined the medical histories for sociodemographic data, surgical approach, body mass index, cut-to-suture time and preoperative diagnosis for THA indication.

Prosthetic stability and cup migration were assessed with EBRA (German: Einzel-Bild-Röntgen-Analyse) [9] from plain X-rays. EBRA is a well-established method that evaluates standard anterior–posterior radiographs without requiring additional means at exposure (e.g., ball markers). Simulating the spatial situation, it computes parameters of longitudinal and transverse migration of prosthetic cup and femoral head. Migration of the femoral head, the acetabular cup and wear in the horizontal and vertical directions can be studied. A comparability algorithm using a grid of transverse and longitudinal tangents of the pelvic contour divides serial radiographs into sets of comparable ones. Migration was measured only between comparable radiographs. The 95% confidence limits for EBRA results are 1.0 mm for longitudinal and 0.8 mm for transverse migration [9].

We followed patients with radiographs before discharge, 6 weeks after surgery and 12 months postoperative. Additional radiographs were performed if the patient had any complaints after THA. All radiographs were taken with the same technique and following the EBRA protocol: anterior–posterior (AP) radiographs; patient standing in upright position and full weight-bearing. For EBRA analysis, a minimum of four radiographs per patient and a minimum radiological follow-up of 6 months was required. Cup migration analysis was performed with EBRA by one independent investigator, who was not involved in the surgeries or the postoperative treatment of the patients. The head and cup sizes used for EBRA calibration were taken from the operation notes.

In addition, the radiographs were checked for radiolucencies according to Delee/Charnley classification [15]. As recommended [16, 17], we divided the patient cohort into two groups to assess the effect of cup size: patients with a cup size < 54 mm and patients with a cup size ≥ 54 mm.

### Statistics

Mean, median, range and standard deviation were calculated for the various measurement parameters. For the analysis, Access and Excel (Microsoft Office Professional Plus 2010, Redmond, WA, USA) as well as Graph Pad Prism (Version 7.0, GraphPad Software, Inc., La Jolla, CA, USA) were used. Total migration was calculated with the Pythagorean theorem expressing the length of the vector. Cup loosening was defined as a total migration of more than 1 mm within 2 years [7]. For comparison of total migration, cup size was first divided into two groups and then the non-parametric Mann–Whitney *U* test was applied. A *p* value of 0.05 was deemed statistically significant.

## Results

A total of 165 cups in 159 patients (female: 90; male: 69) fulfilled our inclusion criteria. In six patients the Pinnacle cup was implanted bilaterally. Patients’ mean age at surgery was 66.7 (range 18.4–90.5) years and mean body mass index was 26.9 kg/m^2^ (range 18.3–50.8). Mean follow-up was 24 (range 7–51) months. Preoperative diagnosis was osteoarthritis in 142 hips (86.1%), avascular necrosis of the femoral head in 22 hips (13.3%) and a fracture of the femoral neck in one hip (0.6%). None of the patients had irradiation. Mean cut-to-suture time was 80 (range 36–209) min. The most frequently used cup sizes in our study were 52 mm (23.6%) and 54 mm (17.6%). None of the implanted cups had screw fixation. Furthermore, only polyethylene and ceramic inlays were used. The most frequently used head size was 32 mm (91.5%). Further details are shown in Tables 1 and 2.

**Table 1:** Demographics of study group

Number of patients	
Female	90
Male	69
Total	159
Mean age (years)	66.7 (18.4–90.5)
BMI (kg/m^2^)	26.9 (18.3–50.8)
Cut-to-suture time (min)	80 (36–209)
Surgical approach	
Direct anterior approach	164
Transgluteal approach	1
Surgical position	
Supine	165
Reaming	
Line-to-line	150
Under-reaming	14
Over-reaming	1
Preoperative diagnosis	
Osteoarthritis	142
Avascular necrosis of the femoral head	22
Fracture of femoral neck	1

Range is given in brackets

**Table 2:** Details of implanted components

Pinnacle acetabular cup size (mm) (%)	
46	1 (0.6)
48	9 (5.5)
50	17 (10.3)
52	39 (23.6)
54	29 (17.6)
56	22 (13.3)
58	24 (14.6)
60	13 (7.9)
62	7 (4.2)
64	3 (1.8)
66	1 (0.6)
Head size (mm) (%)	
28	11 (6.7)
32	151 (91.5)
36	1 (0.6)
Missing data	2 (1.2)

Migration analysis at 24 months follow-up was calculated for 79 of the 165 cups with an EBRA-given comparability limit of 3.0 mm (95% confidence interval). Negative horizontal migration values were defined as medial migration. Negative vertical migration (distal migration) up to 1 mm was caused by the limited accuracy of the EBRA measurement method. For EBRA migration analysis we defined a minimum of four postoperative X-rays of each cup as requisite. None of our patients had to be excluded from EBRA migration analysis. A complete set of radiographs at every measurement point (e.g. 6 months, 12 months, etc.) was not available for each cup in our study. Therefore, total migration could not be calculated in all cases. This gives a different number of cases at the corresponding migration behaviour analysis over time.

**Fig. 1:**
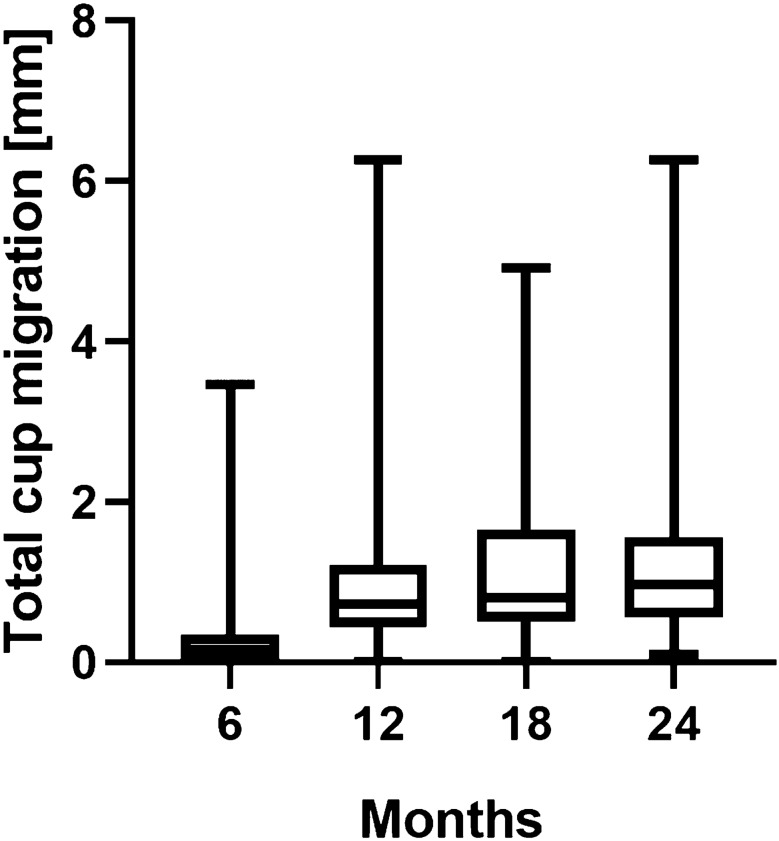
Boxplots showing mean migration and bars showing minimum and maximum migration values for the radiological follow-up

Of the 165 cups 79 had sufficient EBRA follow-up to assess migration behaviour after 2 years. Based on the definition of aseptic loosening, 37 (46.8%) of the 79 showed more than 1 mm migration after 2 years. Table 3 and Fig. 1 give details of migration behaviour after 6, 12, 18 and 24 months. Cup migration in percent is given in Table 4.

**Table 3:** Mean total migration in millimeters (mm) over time

	6 months (*n* = 156)	12 months (*n* = 70)	18 months (*n* = 58)	24 months (*n* = 79)
Migration of the Pinnacle cup in mm (range)	0.3 (0.0–3.5)	1.0 (0.0–6.3)	1.1 (0.0–4.9)	1.2 (0.1–6.3)

**Table 4:** Total migration in millimeters (mm) over time

Total migration (mm)	6 months (*n* = 156)	12 months (*n* = 70)	18 months (*n* = 58)	24 months (*n* = 79)
≤ 1	152 (97.5%)	46 (65.7%)	37 (63.8%)	42 (53.2%)
> 1	3 (1.9%)	19 (27.2%)	12 (20.7%)	28 (35.4%)
> 2	0 (0.0%)	3 (4.3%)	6 (10.3%)	5 (6.3%)
> 3	1 (0.6%)	1 (1.4%)	1 (1.7%)	2 (2.5%)
> 4	0 (0.0%)	0 (0.0%)	2 (3.5%)	1 (1.3%)
> 5	0 (0.0%)	1 (1.4%)	0 (0.0%)	1 (1.3%)

**Fig. 2:**
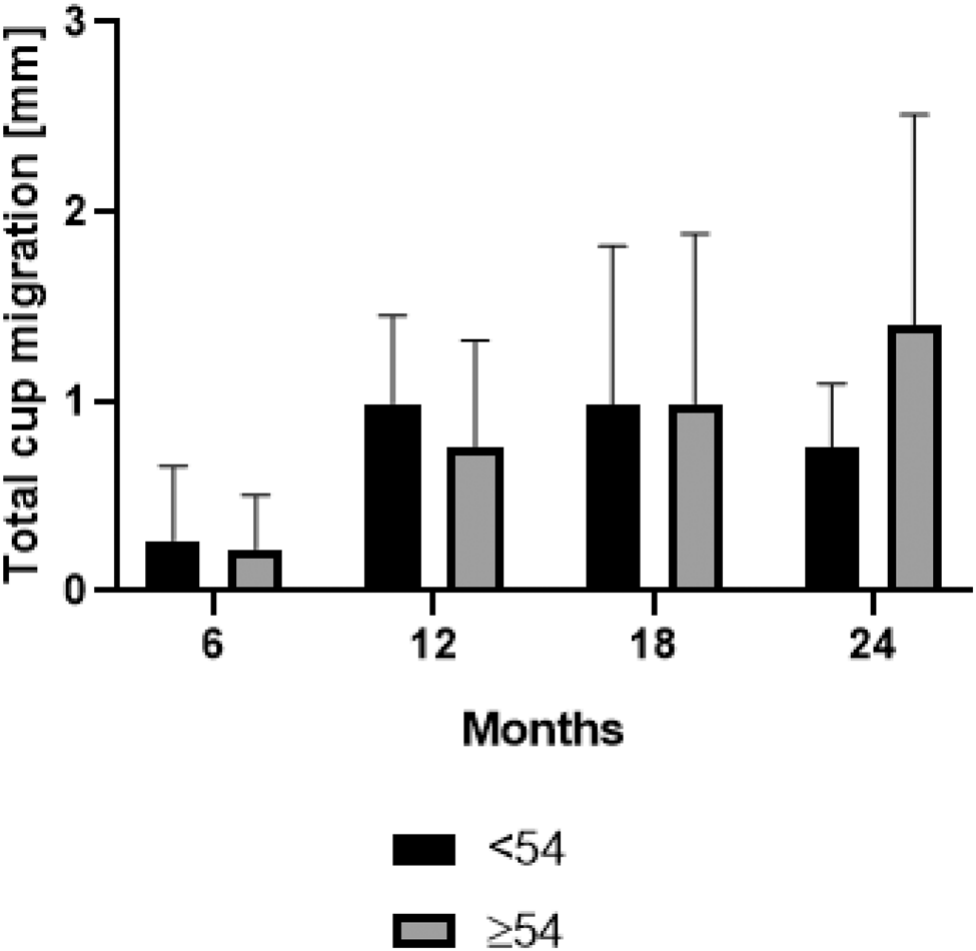
Mean and standard deviation (bars) of total cup migration in patients with cup size < 54 mm and patients with cup size ≥ 54 mm for the clinical follow-up time of 2 years

9 (5.5%) of 165 implants showed radiolucencies over time in at least one of the three zones classified according to Delee/Charnley [15]. The migration of these implants was not significantly increased (*p* > 0.05). Furthermore, no statistically significant difference in total migration was found between the two sub-cohorts: cup size < 54 mm and cup size ≥ 54 mm for 6 months (*p* = 0.9378), 12 months (*p* = 0.1120) and 18 months (*p* = 0.9918). A statistically significantly greater mean total migration was found for cup size ≥ 54 mm after 24 months (*p* = 0.0256) radiological follow-up. At 24 months cup size ≥ 54 mm showed a mean migration of 1.4 (SD 1.1) in comparison to a mean migration of 0.7 (SD 0.3) for cup size < 54. Migration in larger cups (≥ 54 mm) was 200% greater than in smaller cup sizes (< 54 mm). Total cup migration of sub-cohorts is shown in Fig. 2.

Four cups (2.4%) had to be revised during our follow-up period. While two cups had to be replaced after eight and 46 months due to recurrent dislocations, one cup was revised in a two-stage revision after 13 months due to chronic periprosthetic joint infection. In another case, the cause of revision was unknown to us, because it was performed at another hospital. In ten (6.1%) cases the inlay and/or femoral head component had to be changed because of acute periprosthetic joint infection in five hips and because of recurrent dislocation in another five hips.

## Discussion

The Pinnacle acetabular cup system is a cementless cup prosthesis that achieves cup fixation by means of the press-fit method. The present study includes one of the largest numbers of Pinnacle cups analyzed by EBRA. We present a low mean total migration of 0.7 mm for the cups investigated during our 24-month follow-up period. The results of our research are comparable to the results of previously published studies.

It has been shown that early migration of cup components is associated with later aseptic loosening [7]. EBRA is an appropriate method for identifying and measuring the migration behaviour of THA components and offers good predictability of acetabular cup survival based on the assumption that primary stability is a prerequisite for bony ingrowth of the acetabular component [9, 18, 19]. While radiostereometric analysis (RSA) is considered the gold standard for migration measurements, Abrahams et al. recently reported good agreement between EBRA-Cup and RSA measurements for migration of acetabular cups in THA [10]. Consequently, we used the EBRA-Cup software for our investigations of migration behaviour in this retrospective study. The advantage of EBRA over RSA is that EBRA is a non-invasive method.

Stihsen et al. [20] showed a mean total migration of 1.1 mm for the Pinnacle acetabular cup after 2 years follow-up in 57 Pinnacle cups. Given the established threshold of > 1 mm within 2 years as significant migration of an uncemented cup, Stihsen et al. found that 40.4% of their cups migrated more than 1 mm in the observed 2-year follow-up [20]. Our results with cup migration are well in line with the results published by Stihsen et al. [20]. We can report a mean total migration of 1.2 mm after 2 years, with 46.8% of our cups having migrated > 1 mm at that time. However, our cup cohort was larger than that of Stihsen et al.

Several factors and the influence of migration were reported in previous studies. Especially the effect of cup size on migration yielded different results. While Stöckl et al. found no correlation between cup size and migration, Takatori et al. reported a negative correlation between cup size and migration distance [17, 21]. While we did not find any significant differences at 6, 12 or 18 months, a statistically significant higher mean total migration was found for cup size ≥ 54 mm after 24 months (*p* = 0.0256) radiological follow-up. In our study, larger cups (≥ 54 mm) showed 200% greater migration than did smaller cup sizes (< 54 mm). With our results at 24 months we can confirm the findings of Stihsen et al. for the Duraloc cup ‘100 Series’ (DePuy Synthes, Warsaw, IN, USA), who also found a significantly higher migration for cups ≥ 54 mm [16]. Stihsen et al. already suspected that the soft bone surrounding the larger cup provides a larger surface for movement, which may lead to increased migration [16].

Stoeckl et al. [21] investigated and published the migration behaviour of the Duraloc cup ‘100 Series’ (DePuy Synthes, Warsaw, IN, USA) after 2 and 4 years follow-up. The Duraloc cup was the predecessor of the Pinnacle cup system. Using the same criteria for migration analysis, 48% of the Duraloc cups showed significant migration and a mean total migration rate of 1.13 mm at 2 years. However, the reduction in migration rate observed by Stoeckl et al. after 4 years provided a better result for the Duraloc cup than the initially surveyed migration rate after 2 years had suggested [22]. Stihsen et al. also investigated the migration behavior of the Duraloc cup, with 25% of cups considered loose at 2 years and 10% at 4 years [16]. One mechanism that might explain the reduction in radiologically loose cups between 2 and 4 years is secondary stabilization. Already in 1999, Krismer et al. described this phenomenon for stems and came to the conclusion that this migration behavior is not atypical and can lead to long-lasting survival of the implant [23]. Based on the previously mentioned studies and the observed reduction in migration in our study between 1 and 2 years postoperatively, secondary stabilization and osseointegration of the Pinnacle cup can be assumed.

This study has several limitations, including the retrospective methodology and the absence of a control group. Second, patient follow-up was not blinded or randomized, and therefore bias and confounders are difficult to rule out. In addition, other factors that might influence cup migration behavior, such as comorbidities and under-, line-to-line- or over-reaming were not investigated. Third, there were various numbers of radiographs and duration of follow-up for each hip. This may have influenced the migration results due to the smoothing function within the software. Fourth, the EBRA cup software uses the horizontal line that may be labeled on the pubic symphysis or the ischial foramen as the reference segment for proximal translation, while assuming that the pelvis is in continuity and is a single reference segment. Furthermore, some patient characteristics (e.g. smoking, osteoporosis), which might have influenced clinical outcome of the implant, could not be assessed. In addition, we did not perform an inter-rater reliability calculation.

## Conclusion

In conclusion, we showed a low mean total migration rate for the Pinnacle acetabular cup with good clinical results up to 2 years after surgery. Based on the assumption of secondary stabilization and the observed reduction in migration over time, we expect a good long-term result. Further investigations and a longer follow-up are needed to confirm these expectations. In addition, there is a need for further research in regard to osteointegration.

## Data Availability

Data will be sent if necessary.
